# Oral mannitol for bowel preparation: a dose-finding phase II study

**DOI:** 10.1007/s00228-022-03405-z

**Published:** 2022-10-26

**Authors:** Cristiano Spada, Giancarla Fiori, Peter Uebel, Gian Eugenio Tontini, Paola Cesaro, Leonardo Minelli Grazioli, Pietro Soru, Ivana Bravi, Carsten Hinkel, Alberto Prada, Dhanai Di Paolo, Tim Zimmermann, Gianpiero Manes, Jean Christophe Valats, Ralf Jakobs, Luca Elli, Marino Carnovali, Giorgio Ciprandi, Franco Radaelli, Maurizio Vecchi

**Affiliations:** 1 U.O. Endoscopia Digestiva, Fondazione Poliambulanza – Istituto Ospedaliero, Brescia, Italy; 2 Divisione Di Endoscopia, Istituto Europeo Di Oncologia, IRCCS, Milan, Italy; 3 Praxis Für Gastroenterologie Und Fachärztliche Innere Medizin, Im Haus Der Gesundheit, Ludwigshafen am Rhein, Germany; 4 Department of Pathophysiology and Organ Transplantation, University of Milan, Milan, Italy; 5 Gastroenterology and Endoscopy Unit, IRCCS Fondazione Ca’ Granda Ospedale Maggiore Policlinico, Via F. Sforza 35, 20100 Milan, Italy; 6 Servizio Gastroenterologia Ed Endoscopia Digestiva, Istituto Auxologico Italiano, Milan, Italy; 7 U.O.C. Gastroenterologia, Ospedale Valduce, Como, Italy; 8 Klinikum Worms Medizinische Klinik II, Worms, Germany; 9 U.O.C. Gastroenterologia, ASST Rhodense, Presidi Di Rho E Garbagnate, Garbagnate Milanese, MI Italy; 10 Hépato-Gastro-Entérologie, Centre Hospitalier Universitaire de Montpellier, Montpellier, France; 11 Medizinische Klinik C, Klinikum der Stadt Ludwigshafen, Ludwigshafen, Germany; 12 Ospedale Papa Giovanni XXIII, Bergamo, Italy; 13 Casa Di Cura Villa Montallegro, Genoa, Italy

**Keywords:** Colonoscopy, Bowel preparation, Mannitol, Dose-finding, Phase II randomized trial

## Abstract

**Background:**

Successful bowel preparation (BP) for colonoscopy depends on the instructions, diet, the laxative product, and patient adherence, which all affect colonoscopy quality. Nevertheless, there are no laxatives which combine effectiveness, safety, easy self-administration, good patient acceptance, and low cost. However, mannitol, a sugar alcohol, could be an attractive candidate for use in clinical practice if it is shown to demonstrate adequate efficacy and safety.

**Aims:**

The present phase II dose-finding study compared three doses of mannitol (50, 100, and 150 g) to identify the best dose to be used in a subsequent phase III study.

**Methods:**

The Boston Bowel Preparation Scale, caecal intubation rate, adherence, acceptability, and safety profile, including measurement of potentially dangerous colonic gas concentrations (CH_4_, H_2_, O_2_), were considered in all patients. A weighted algorithm was used to identify the best mannitol dose for use in the subsequent study.

**Results:**

The per-protocol population included 60 patients in the 50 g group, 54 in the 100 g group, and 49 in the 150 g group. The 100 g dose was the best as it afforded optimal colon cleansing efficacy (94.4% of patients had adequate BP), adherence, acceptability, and safety, including negligible gas concentrations.

**Conclusions:**

The present study demonstrated that the colon cleansing efficacy and safety of mannitol were dose dependent. Conversely, gas concentrations were not dose dependent and negligible in all patients. Combined evaluation of efficacy, tolerability, and safety, using a weighted algorithm, determined that mannitol 100 g was the best dose for the phase III study.

## Introduction

Colonoscopy is the gold standard for the inspection of colonic mucosa and so is currently widely used for symptom diagnosis, surveillance, and colorectal cancer screening [1]. The quality of colonoscopy is critically affected by bowel preparation (BP), which is a multifaceted procedure encompassing instructions, diet, laxative products, and patient compliance [2]. Moreover, the quality of colonic cleansing significantly affects diagnostic accuracy, timing, and completeness [3]. Consequently, inadequate BP can result in missed lesion detection, increased complications, unnecessarily prolonged examinations, the need for repeat BP, and additional patient discomfort [4].

The suitability of BP products is influenced by several factors, both product related (taste, volume, intake, schedule) and patient related (age, acceptance, comorbidity, concomitant medications). In addition, the lack of standardized patient instructions is associated with inadequate BP [2, 5] even though patient adherence is extremely important for achieving adequate bowel cleansing [5].

The European Society of Gastrointestinal Endoscopy (ESGE) guidelines recommend osmotic laxatives for BP [6]. Polyethylene glycol (PEG) is the most commonly used product in Europe. High-volume (4 L) PEG has long been considered the reference standard for BP due to its recognized efficacy [7]. However, a sizeable number of patients find it unpleasant and do not take the recommended amount due to its bad taste, large volume, or feelings of nausea [8]. Therefore, other preparations have been developed to reduce the drawbacks associated with PEG electrolyte lavage solution (ELS). PEG ascorbate (PEG ASC) requires the ingestion of only 2 L of preparation, has a better taste, and thus is more tolerated than the original PEG formulation [9]. Nonetheless, inadequate BP still affects 20–25% of all colonoscopies [10].

Consequently, the ideal laxative for colonoscopy should demonstrate a combination of effectiveness, safety, easy self-administration, good patient acceptance, and low cost [11]. None of the available laxatives meet all these requirements, but mannitol could be an attractive candidate.

Mannitol is a sugar alcohol only minimally absorbed following oral administration. It acts as an osmotic laxative by increasing osmolarity in the gut. Consequently, the amount of fluid retained in the bowel increases and the entire content of the colon may be excreted [12]. Mannitol was widely used in the late 1970s and early 1980s as a bowel cleansing prep agent for colonoscopy due to its effectiveness, easy self-administration because of reduced volumes, pleasant taste, and lack of significant systemic side effects. Indeed, the most frequently reported adverse events (AEs) were mild serum electrolyte changes, nausea, and abdominal pain, all of which are self-resolving. Other AEs were vomiting, abdominal distension, and increased haematocrit. Notably, general safety and tolerability were comparable between mannitol and PEG formulations. All published studies used doses of mannitol of between 50 and 150 g, given in a single dose, except for one study which tested a split dose of mannitol solution [13].

Two studies demonstrated that mannitol at the dose of 50 g showed the same effectiveness as other bowel cleansing agents [14, 15]. Moreover, another trial showed that the 50 g split-dose patients tolerated mannitol better as regards overall experience, nausea, post-procedure discomfort, disagreeable flavour, volume ingested, and cost [13]. Mannitol at 100 g showed comparable efficacy to several bowel cleansing agents, including sodium phosphate, and 4 L and 2 L PEG, and essentially had a similar incidence of AEs [16–20]. Finally, mannitol at the dose of 150 g was as effective as sodium picosulphate with a similar safety profile [21].

Currently, mannitol is commonly used in Brazil, and, although off-label, is still the most popular formulation for bowel cleansing in that country.

In Europe and the USA, mannitol is not currently used for bowel cleansing due to anecdotal reported cases of intestinal explosion (one lethal) which were attributed to a mixture of methane (CH_4_), hydrogen (H_2_), and oxygen (O_2_) during diathermy-electrocautery after biopsy [22, 23]. However, as no controlled studies have been performed to clarify this issue, mannitol should be re-examined in a randomized and controlled trial.

Consequently, a randomized clinical trial was planned with an adaptive design consisting initially of a dose-finding phase (phase II) followed promptly by a non-inferiority study versus a reference drug study (phase III) using the optimal mannitol dose as determined in the dose-finding phase. The results of the dose-finding phase (phase II) are reported here.

## Materials and methods

### Study design

The dose-finding phase II trial was designed as an international, multicentre, randomized, parallel-group, endoscopist-blinded study. The endoscopists performing study colonoscopies were blinded to treatment dose assignment. Separate, unblinded investigators were responsible for assigning, allocating, and reporting study treatment.

The study was conducted in three countries: Italy, Germany, and France. Nine sites were involved: six in Italy, two in Germany, and one in France. The ethics committee (EC) of the primary centre approved the study on 12 February 2020 (EC MI-AREA 3; No. 62-12022020), followed by the ECs of the other centres. The study was registered on EudraCT (2019-002856-18).

The study investigated three mannitol doses: 50 g, 100 g, and 150 g. The expected sample size was 50 patients for each dose. Thus, 150 adult patients who had provided written informed consent (visit 1) and had fulfilled all eligibility criteria (visits 1 and 2) were scheduled for elective (screening, surveillance, or diagnostic) colonoscopy and were randomized (visit 3) in a 1:1:1 ratio to the three dosage groups. Randomization was stratified by centre and the presence of constipation (yes/no), defined as recurrent use of laxatives or a Bristol Stool Form Scale < 3 in the 2 weeks before randomization [24].

Before administration, mannitol powder was dissolved in water at room temperature: 50 g of mannitol powder in 500 mL of water, 100 g in 750 mL, and 150 g in 1000 mL. Patients had to drink the solution on the day of colonoscopy within 30 min for the 50 and 100 g doses and 60 min for the 150 g dose; they had to complete administration at least 4 h before the colonoscopy. In addition, patients were to drink about 1 L of clear liquid in the next hour to prevent dehydration. The patient’s completion of BP was evaluated through a form filled in by the patient on the morning of the day of colonoscopy (visit 4). Patients were also assessed for safety and preparation tolerability at visit 4.

OPIS (Desio, Italy), a contract research organization, was employed to manage all phases of the trial.

### Eligibility criteria

The inclusion criteria were as follows: ability of the patient to consent and provide signed written informed consent, age ≥ 18 years, patient scheduled for elective (screening, surveillance, or diagnostic) colonoscopy to be prepared and performed according to ESGE guidelines, and patient willing and able to complete the entire study and to comply with instructions. The main exclusion criteria were as follows: pregnancy or breast feeding, severe renal failure (eGFR < 30 ml/min/1.73 m^2^), severe heart failure (NYHA Class III–IV), severe anaemia (Hb < 8 g/dl), severe acute and chronically active inflammatory bowel disease, chronic liver disease (Child–Pugh class B or C), electrolyte disturbances, recent (< 6 months) symptomatic acute ischaemic heart disease, history of significant gastrointestinal surgery, and use of laxatives, colon motility-altering drugs, and/or other substances.

### Criteria for dose evaluation

The dose selection process used a weighted algorithm considering bowel cleansing efficacy, safety regarding colonic gas concentrations, tolerability profile, adherence, and acceptability (Tables 1 and 2).

**Table 1:** Criteria for clinical judgement to determine the most appropriate dose

**Criteria**		**Scores**		
		**Best result**	**Intermediate result**	**Worst result**
A.	Rate of adequate bowel cleasing	6	3	0
B.	Rate of patients in a safe condition	4	2	0
C.	Clinical judgement score	2	1	0

**Table 2:** Sub-criteria for clinical judgement to determine the most appropriate dose

**Sub-criteria for clinical judgement score**	**Scores**		
	**Best result**	**Intermediate result**	**Worst result**
C1. Rate of patients with confirmed caecal intubation	2	1	0
C2. Rate of patients without related AEs	2	1	0
C3. Rate of patients with adherence to study drug administration	2	1	0
C4. Mean ease of use (NRS)	2	1	0
C5. Mean taste (NRS)	2	1	0
C6. Rate of patients willing to reuse the study preparation	2	1	0

*AE* adverse event, *NRS* numeric rating scale

#### Bowel cleansing

Adequate bowel cleansing was evaluated by the blinded endoscopist and defined as a Boston Bowel Preparation Scale (BBPS) total score of ≥ 6, with a minimum score for each of the three colon segments of ≥ 2 during colonoscopy after standard washing and air insufflation for luminal distension [25]. The other efficacy parameter was caecal intubation rate, defined as the percentage of patients with the appendiceal orifice visible to the endoscopist.

The efficacy of the preparation as a bowel cleansing agent was evaluated by the blinded endoscopist by determining the BBPS score for each of the three colon segments (right: transverse, including flexures; and left: including sigmoid and rectum) during colonoscopy.

#### Safety

The safety of mannitol was assessed according to:

- The proportion of patients in a safe condition, defined as the absence in each colon segment of potentially dangerous H_2_ and/or CH_4_ (≥ 4.0% vol and ≥ 5.0% vol, respectively) concentrations during colonoscopy after standard washing and air insufflation for luminal distension. Intestinal H_2_, CH_4_, and O_2_ concentrations were measured in each colon segment using a multi-gas detector (Dräger X-am^®^ 8000, Dräger Italia, Milan, Italy). Of note, carbon dioxide (CO_2_) insufflation and water-aided techniques for colon distension (e.g., water immersion and water exchange) were not permitted. The gas detector had no direct contact with the patient’s body but was connected through a one-way pump and a filter to a polyvinyl catheter inserted into the working channel of the colonoscope. The intestinal gases were conveyed to the gas detector by a one-way pump that prevented the return of gases to the colonoscope. Electrocautery devices were allowed only during withdrawal and after washing and air insufflation as electrosurgical procedures must be avoided in case H_2_ and/or CH_4_ levels are dangerous.

#### Tolerability

Tolerability was assessed according to:

- The incidence of AEs from the beginning of the administration of the study drug (i.e., treatment-emergent adverse events, TEAEs). AEs were reported and monitored according to good clinical practice guidelines. TEAEs related to the study drug (i.e., AEs that developed or worsened in severity on or after mannitol administration and judged to be related to the drug by the investigator) that occurred during the study were considered.
- The proportion of patients demonstrating a change from baseline, considered clinically significant by the investigator, in haematological and chemistry parameters 4 h and 8 h after completion of study drug administration, where clinically significant meant that the change required an additional control or medical intervention.
- The proportion of patients with a change in vital signs (heart rate and pulse oximetry) during colonoscopy, considered clinically significant by the investigator, where clinically significant meant that the change required an additional control or medical intervention.

#### Adherence

Adherence was defined as the study drug was completely taken, partially taken, or not taken.

#### Acceptability

Acceptability was defined by:

- Ease of use (assessed by a numeric rating scale (NRS): 0 = very difficult to 10 = very easy)
- Taste (NRS: 0 = terrible to 10 = very good)
- Willingness to reuse the preparation (yes/no).

#### Algorithm for dose selection

The appropriate mannitol dose to be used in the comparative non-inferiority phase (phase III) was chosen at the end of the dose-finding study based on a weighted algorithm considering the following criteria:

- A, rate of adequate bowel cleansing (at least 75%, otherwise the dose was not considered for selection of the optimal dose)
- B, rate of patients in a safe condition, defined as the absence of potentially dangerous levels of H_2_ and CH_4_ in each colon segment after standard washing and air insufflation for luminal distension
- C, clinical judgement based on the caecal intubation rate, the incidence of AEs related to the study drug, treatment adherence, and acceptability (considering ease of use, taste, and willingness to reuse the preparation).

A score was assigned to each treatment group according to the group’s ranking for each of the three main criteria (A, B, and C), in keeping with the principle ‘the higher, the better’, as reported in Table 1. The clinical judgement score for each treatment group was the sum of the sub-scores given to the sub-criteria according to the following scheme and the principle ‘the higher, the better’, as reported in Table 2. If two or all three doses proved to be equally safe and effective, the lowest dose was selected for phase III of the study. The dose for phase III was to be selected as the minimum dose with the highest total score, computed as the sum of the scores given to each main criterion.

#### Data collection

Designated investigator staff entered the data required by the protocol in an electronic case report form (eCRF) using fully validated software. On-line validation programmes checked for data discrepancies and allowed the data to be confirmed or corrected before transfer to the contract research organization by generating appropriate error messages. Finally, the database was locked after all the above actions had been finished, and the database was declared complete and accurate.

### Statistical analysis

#### Sample size determination

The sample size was based on the precision of the estimate within each treatment group, i.e., the 95% confidence interval of the proportion of patients in each treatment group with adequate bowel cleansing (BBPS total score ≥ 6, with BBPS ≥ 2 for each segment).

Table 3 shows the precision of the estimate for different rates of adequate bowel cleansing with a sample size of 50 patients.

**Table 3:** Correlation between rate of adequate bowel cleansing and precision of the estimate within each treatment group

**Rates of adequate bowel cleansing (%)**	Estimate precision (%) (i.e., 95% CI)*
75	±12
80	±11
85	±10

^*^Confidence interval estimated through normal approximation of binomial distribution without continuity correction

In order to limit patient exposure to an ineffective dose necessitating a repeat colonoscopy, the adequacy of bowel cleansing would be monitored continuously, and enrolment in a treatment group would be discontinued as soon as 25% of treated patients presented inadequate bowel cleansing and required a repeat colonoscopy.

#### Analysis populations

The following populations would be used for the statistical analyses:

- Safety set: all patients who took the study preparation, even partially
- Modified safety set: all patients who took the study preparation, even if only partially, and who did not significantly fail to meet inclusion/exclusion criteria
- Full analysis set (FAS): all randomized patients who took the study preparation, even if only partially, underwent a colonoscopy and had a BBPS available for at least one colon segment after standard washing and air insufflation for luminal distension
- Per protocol (PP): all randomized patients who met the following criteria:
  - Treatment with the study drug completed
  - Colonoscopy completed adequately in the absence of pathological obstruction that prevented access to the right colon, including the cecum (i.e., the endoscope did not meet obstacles other than faecal material), and without acute deterioration of the patient’s general condition causing suspension of the procedure
  - BBPS and H_2_ and CH_4_ measurements were available for all colon segments after standard washing and air insufflation for luminal distension
  - No significant protocol violations regarding inclusion/exclusion criteria or that could impact evaluations.

#### Descriptive statistics

Standard descriptive statistics (i.e., the mean, standard deviation, median, minimum and maximum, 1st and 3rd quartiles) were used to summarize continuous data. Frequencies and percentages were used to summarize categorical data. Logistic regression was used to evaluate the difference between groups. All analyses were performed using SAS^®^ release 9.4 or later (SAS Institute, Inc., Cary, NC, USA).

## Results

### Patient disposition

**Fig. 1:**
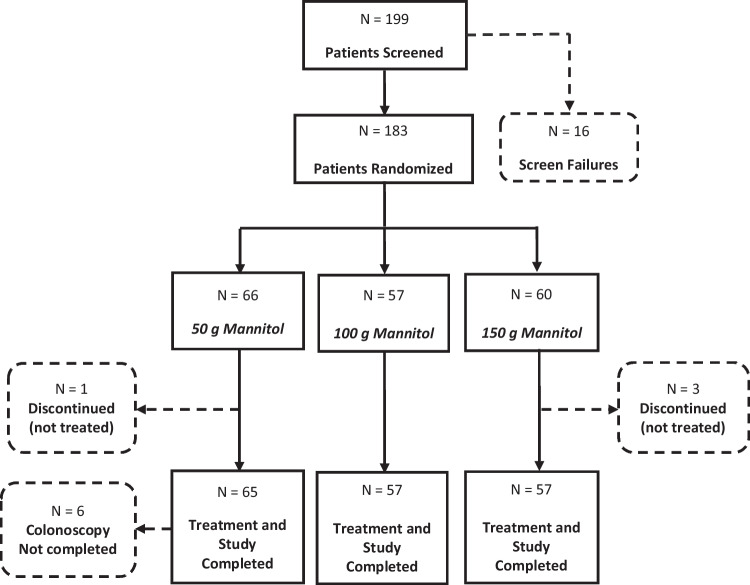
Patients’ disposition (randomized set)

Investigators screened 199 patients and randomized 183 patients. A total of 179 patients completed the study, of whom 163 were considered for the per-protocol (PP) analysis (Fig. 1).

All 179 randomized patients underwent colonoscopy and had the bowel cleansing of at least one colon segment assessed (BBPS). All patients in the PP population completed colonoscopy except for five patients in the 50 g mannitol group due to inadequate bowel cleansing.

The demographic and clinical characteristics of the PP population are reported in detail in Table 4.

**Table 4:** Demographic characteristics (PP population)

	**50 g mannitol** **(*N* = 60)**	**100 g mannitol** **(*N* = 54)**	**150 g mannitol** **(*N* = 49)**	**Total** **(*N* = 163)**
Age at study entry (years)				
*n*	60	54	49	163
Mean (SD)	57.3 (11.41)	54.4 (11.47)	54.4 (12.37)	55.5 (11.74)
Median	57.0	55.0	55.0	56.0
Q1; Q3	49.0; 66.0	48.0; 61.0	46.0; 64.0	48.0; 64.0
Min; Max	28; 81	25; 75	26; 78	25; 81
Sex, *n* (%)				
Male	31 (51.67)	25 (46.30)	18 (36.73)	74 (45.40)
Female	29 (48.33)	29 (53.70)	31 (63.27)	89 (54.60)
Ethnicity, *n* (%)				
Hispanic or Latino/a	1 (1.67)	0	2 (4.08)	3 (1.84)
Not Hispanic or Latino/a	58 (96.67)	51 (94.44)	46 (93.88)	155 (95.09)
Unknown	1 (1.67)	3 (5.56)	1 (2.04)	5 (3.07)
Female reproductive status^a^, *n* (%)	29	29	31	89
Childbearing potential	8 (27.59)	6 (20.69)	13 (41.94)	27 (30.34)
Menopause	19 (65.52)	20 (68.97)	18 (58.06)	57 (64.04)
Infertile	2 (6.90)	3 (10.34)	0	5 (5.62)

Percentages were computed on patients belonging to the Full analysis set

‘Unknown’ ethnicity refers to undetermined ethnicity

*Q1* 1st quartile, *Q3* 3rd quartile, *SD* standard deviation

^a^Percentages were calculated on female patients belonging to the full analysis set

### Bowel cleansing

Mannitol proved to be an effective bowel cleansing agent as the minimum rate of adequate bowel cleansing (75%) necessary for selection of a dose for phase III of the study was reached in all treatment groups. In fact, 75% of patients in the 50 g group, 94.44% in the 100 g group, and 93.88% in the 150 g group presented an adequate level of bowel cleansing during colonoscopy (Table 5). In addition, the BBPS sub-score for each segment of the colon was ≥ 2 for 94–98% of the patients in the 100 g and 150 g dose groups and for 82–83% of those in the 50 g group.

**Table 5:** Summary of BBPS scores and adequate bowel cleansing (PP population)

	**50 g mannitol** **(*N* = 60)**	**100 g mannitol** **(*N* = 54)**	**150 g mannitol** **(*N* = 49)**
Right colon BBPS sub-score, *n* (%)			
< 2	11 (18.33)	3 (5.56)	3 (6.12)
≥ 2	49 (81.67)	51 (94.44)	46 (93.88)
Transverse colon BBPS sub-score, *n* (%)			
< 2	10 (16.67)	1 (1.85)	1 (2.04)
≥ 2	50 (83.33)	53 (98.15)	48 (97.96)
Sigmoid–rectal junction colon BBPS sub-score, *n* (%)			
< 2	11 (18.33)	2 (3.70)	2 (4.08)
≥ 2	49 (81.67)	52 (96.30)	47 (95.92)
Adequate bowel cleansing, *n* (%)			
No	15 (25.00)	3 (5.56)	3 (6.12)
Yes	45 (75.00)	51 (94.44)	46 (93.88)
Percentage of patients with adequate bowel cleansing (95% CI)	75% (0.64–0.86)	94% (0.88–1)	94% (0.87–1)

Percentages were computed on patients belonging to the PP population

A patient had adequate bowel cleansing if BBPS total score was ≥ 6, with a score for each of the three colon segments of ≥ 2

If at least one sub-score was not available (due to missed colonoscopy completion because of inadequate bowel cleansing), the patient was considered not having adequate bowel cleansing

A 95% Wald Confidence Interval for the percentage was provided for each dose level

*BBPS* Boston Bowel Preparation Scale, *PP* per protocol

**Fig. 2:**
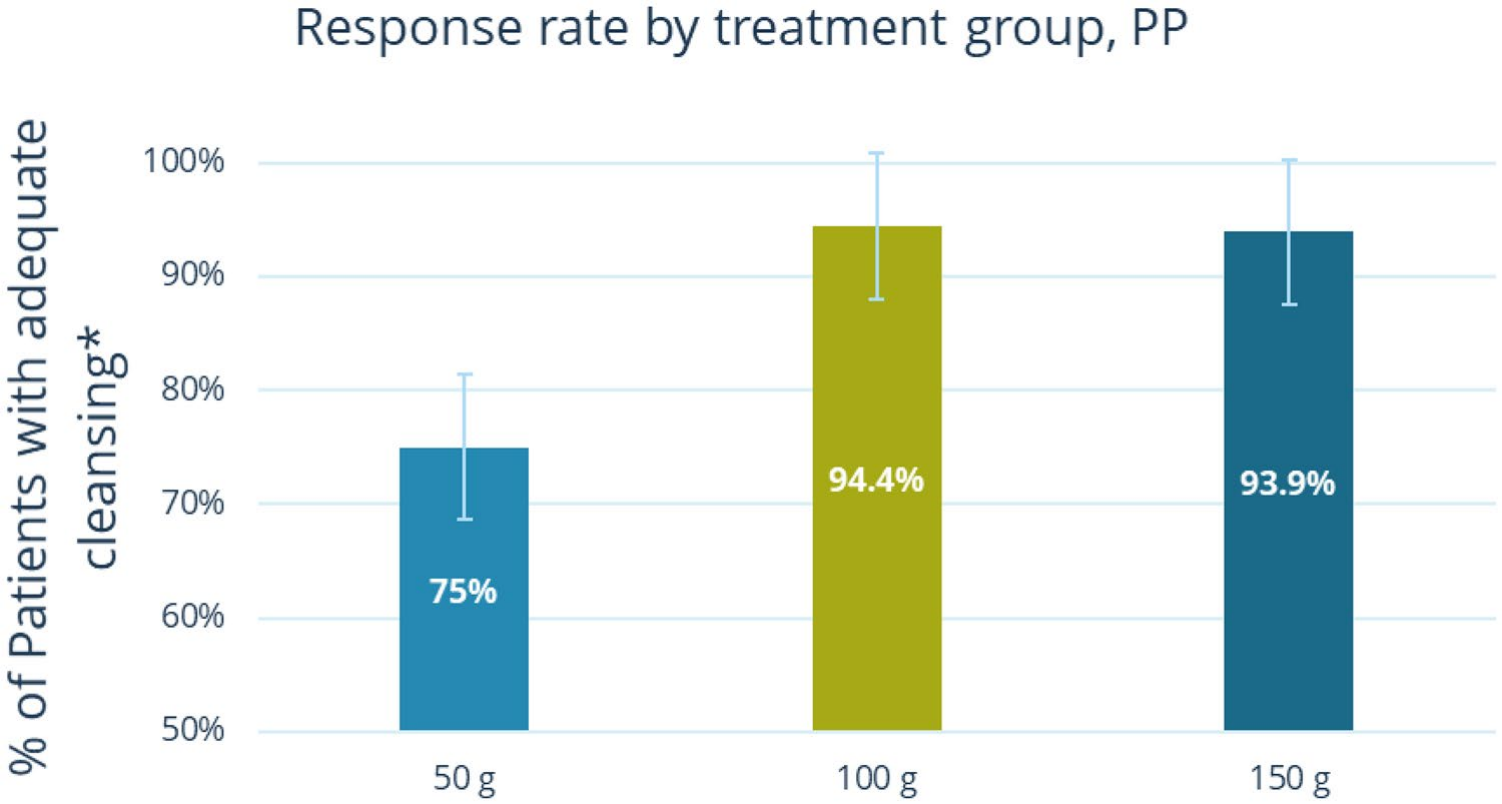
Response rates by treatment group (PP population). *A patient has adequate bowel cleansing if Boston Bowel Preparation Scale (BBPS) total score ≥ 6, with a score for each of the three colon segments ≥ 2. In case of at least one sub-score not available (due to the missed completion of the colonoscopy due to inadequate bowel cleansing), the patient was considered not having adequate bowel cleansing. BBPS, Boston Bowel Preparation Scale; CI, confidence interval; PP, per protocol

Dose and response were found to be significantly correlated. The logistic regression model showed a statistically significant correlation between administered dose and response (*χ*^2^ = 10.6690, *P* = 0.0048), as reported in Fig. 2. In particular, a statistically significant difference in the rate of patients with adequate bowel cleansing between the 100 g and 50 g groups (OR 5.665; 95% CI: 1.540–20.841; *P* = 0.0091) and the 150 g and 50 g groups (OR 5.111; 95% CI: 1.385–18.862; *P* = 0.0143) was observed, thus indicating that both the 100 g and 150 g dose groups had a higher proportion of patients with adequate bowel cleansing than the 50 g group. On the other hand, the difference between the 100 g and 150 g dose groups was not statistically significant (OR 0.902; 95% CI: 0.173–4.693; *P* = 0.9026), thus confirming that the rate of adequate bowel cleansing increased with increasing mannitol dose, reaching a maximal response plateau with the 100 g dose (i.e., no further increase in the rate of adequate bowel cleansing was observed at a higher dose, i.e., 150 g). The rate of confirmed caecal intubation for the PP population was 91.67% in the 50 g group and 100% in the 100 g and 150 g mannitol groups. These results were confirmed by controlling for the two stratification factors at randomization (study centre and presence of constipation in the 2 weeks before randomization) and by a model assessing the influence of potential prognostic factors (i.e., age and number of previous unsuccessful bowel cleansing procedures) on dose response.

### Adherence

Treatment adherence was excellent. All patients in the 50 g, 100 g, and 150 g dose groups fully adhered to taking mannitol as prescribed (Table 6).

**Table 6:** Dose selection using the weighted algorithm (PP population)

	**50 g mannitol (*N* = 60)**	**100 g mannitol (*N* = 54)**	**150 g mannitol (*N* = 49)**
Summary results			
A: Rate of adequate bowel cleansing (%)	75	94.44	93.88
B: Rate of patients in a safe condition (%)	100	100	100
C: Clinical judgement score			
C1: Rate of patients with confirmed caecal intubation (%)	91.67	100	100
C2: Rate of patients without related AEs (%)	96.67	88.89	83.67
C3: Rate of patients with adherence to study drug administration (%)	100	100	100
C4: Mean ease of use (NRS)	9.57	9.28	9.06
C5: Mean taste (NRS)	9.15	8.3	8.02
C6: Rate of patients willing to reuse the study preparation (%)	100	94.44	93.88
Score for single criteria			
A: Rate of adequate bowel cleansing	0	6	3
B: Rate of patients in a safe condition	4	4	4
C: Clinical judgement score			
C1: Rate of patients with confirmed caecal intubation	0	2	2
C2: Rate of patients without related AEs	2	1	0
C3: Rate of patients with adherence to study drug administration	2	2	2
C4: Mean ease of use (NRS)	2	1	0
C5: Mean taste (NRS)	2	1	0
C6: Rate of patients willing to reuse the study preparation	2	1	0
Score for main criteria			
A: Rate of adequate bowel cleansing	0	6	3
B: Rate of patients in a safe condition	4	4	4
C: Clinical judgement score	2	1	0
Total score	6	11	7

**Dose selected**		**X**	

*AE* adverse event, *NRS* numeric rating scale, *PP* per protocol

### Acceptability

As shown in Table 6, the acceptability of mannitol was judged to be very good in all dose groups. As regards ease of use, the NRS score was similar and above 9/10 in all three groups. The mean duration of mannitol intake was 28.4 ± 5.93 min (range: 2–44) in the 50 g dose group, 28.3 ± 4.32 min (range: 15–30) in the 100 g group, and 44.9 ± 16.66 min (range: 15–60) in the 150 g group. Thus, for the highest dose, the mean duration of mannitol intake was significantly shorter than recommended, as the prepared solution of 150 g mannitol was required to be drunk within 60 min on the day of colonoscopy, and highlights the ease of self-administration of the mannitol preparation.

The taste of mannitol was rated as particularly pleasant with an NRS score above 8 for all dose groups; the mean score assigned by patients in the 50 g group was above 9/10.

Furthermore, the percentage of patients willing to reuse mannitol was also very high, being above 90% in all dose groups and 100% for the 50 g group.

### Safety

The mean intestinal concentrations of H_2_ and CH_4_ (% vol) were negligible in all colon segments and similar across treatment groups with no correlation with the dose taken. The mean overall values of H_2_ ranged from 0.02 to 0.25% vol, and those of CH_4_ from 0.00 to 0.04% vol. In all mannitol dose groups and in all colon segments, the mean values of O_2_ concentration (20–21%) corresponded to those of room air and were indicative of effective air insufflation.

No patients in the PP population reported potentially dangerous levels of H_2_ (> 4.0% vol) or CH_4_ (> 5% vol) in any colon segment after standard washing and air insufflation for luminal distension (Table 7).

**Table 7:** H_2_ and CH_4_ mean concentrations in each colon segment (PP population)

	**50 g mannitol**	**100 g mannitol**	**150 g mannitol**
	**(*N* = 60)**	**(*N* = 54)**	**(*N* = 49)**
Right colon			
H_2_ (% vol)			
*n*	57	54	49
Mean (SD)	0.254 (0.6357)	0.108 (0.2148)	0.155 (0.3894)
CH_4_ (% vol)			
*n*	57	54	49
Mean (SD)	0.0430 (0.14375)	0.0250 (0.09253)	0.0122 (0.04150)
O_2_ (% vol)			
*n*	57	54	49
Mean (SD)	20.44 (0.907)	20.31 (1.030)	20.50 (0.990)
Transverse colon			
H_2_ (% vol)			
*n*	57	54	49
Mean (SD)	0.094 (0.2427)	0.061 (0.1493)	0.049 (0.1436)
CH_4_ (% vol)			
*n*	57	54	49
Mean (SD)	0.0228 (0.06414)	0.0102 (0.02639)	0.0051 (0.02337)
O_2_ (% vol)			
*n*	57	54	49
Mean (SD)	20.68 (0.874)	20.51 (0.588)	20.97 (1.605)
Sigmoid-rectal junction colon			

H_2_ (% vol)			
*n*	58	54	49
Mean (SD)	0.019 (0.0664)	0.038 (0.1236)	0.016 (0.0444)
CH_4_ (% vol)			
*n*	58	54	49
Mean (SD)	0.0034 (0.01278)	0.0120 (0.04444)	0.0021 (0.01000)
O_2_ (% vol)			
*n*	58	54	49
Mean (SD)	20.64 (0.641)	20.66 (0.598)	20.93 (1.287)

*PP* per protocol, *SD* standard deviation

### Tolerability

At least one TEAE related to the study drug was reported for 2 out of 65 patients (3.1%) in the 50 g dose group, 6 out of 57 (10.5%) in the 100 g group, and 12 out of 57 (21.1%) in the 150 g group (Table 8).

**Table 8:** Summary of patients with treatment emergent adverse events related to the study drug by MedDRA System Organ Class and Preferred Term (Safety Set)

	**50 g mannitol (*N* = 65)**	**100 g mannitol (*N* = 57)**	**150 g mannitol (*N* = 57)**
	***n* (%)**	***n* (%)**	***n* (%)**
Patients with at least one TEAE related to study drug	2 (3.08)	6 (10.53)	12 (21.05)
MedDRA System Organ Class/Preferred Term			
Gastrointestinal disorders	1 (1.54)	6 (10.53)	11 (19.30)
Nausea	0	4 (7.02)	3 (5.26)
Vomiting	1 (1.54)	2 (3.51)	8 (14.04)
Investigations	1 (1.54)	0	0
Blood creatinine increased	1 (1.54)	0	0
Metabolism and nutrition disorders	0	0	1 (1.75)
Hyperkalaemia	0	0	1 (1.75)
Musculoskeletal and connective tissue disorders	0	0	1 (1.75)
Muscle spasms	0	0	1 (1.75)
Nervous system disorders	0	0	1 (1.75)
Headache	0	0	1 (1.75)
Vascular disorders	0	1 (1.75)	1 (1.75)
Hypotension	0	1 (1.75)	1 (1.75)

Percentages were computed on patients belonging to the safety set

A TEAE is defined as an AE that starts or worsens in severity on or after the mannitol self-administration date. Patients who experienced more than one TEAE were counted only once in each row

Terms were coded using MedDRA, version 22.1

An AE is defined as related to the study drug if a relationship with the study drug is ‘suspected’. The System Organ Class terminology ‘Investigation’ refers to ‘Laboratory investigation’

*AE* adverse event, *TEAE* treatment emergent adverse event

The most frequent TEAEs related to the study drug were vomiting (1 patient in the 50 g dose group, 2 in the 100 g group, and 8 in the 150 g group) and nausea (4 patients in the 100 g group and 3 in the 150 g group).

No deaths were reported, and only one patient in the highest dose group experienced a treatment-emergent serious adverse event (TESAE), which was syncope not related to the study drug.

### Dose selection

The mannitol dose to be used in the comparative non-inferiority phase (phase III) was selected in the PP population based on the weighted algorithm (Table 6).

The total score was 11 for the 100 g dose, 7 for the 150 g dose, and 6 for the 50 g dose, and therefore 100 g was identified as the mannitol dose to be used in the comparative non-inferiority phase III study.

Results for the FAS were consistent with those of the PP population and thus further supported the selection of 100 g mannitol for phase III.

## Discussion

A low-volume bowel cleansing agent with good palatability and ideally administered shortly before the colonoscopy procedure, and which also demonstrates good patient compliance, thus increasing procedure success rates, is currently not available. However, mannitol, a sugar alcohol only minimally absorbed following oral administration that acts as an osmotic laxative by increasing osmolarity in the gut, is a possible candidate for such an agent.

The purpose of this phase II dose-finding study was to identify the most appropriate dose of mannitol for BP to be used in phase III of the study.

The present study demonstrated that mannitol was an effective bowel cleansing agent at all studied doses. However, the 100 g dose was the most effective, especially regarding cleansing efficacy.

The weighted algorithm, specifically designed for this study, consistently confirmed the intermediate dose as the optimal choice. The balanced comparison of different characteristics by the algorithm identified the 100 g dose as the ideal concentration to effectively clean the colon in more than 90% of patients. It also had an excellent safety profile, including the absence of potentially dangerous levels of intestinal gas, and was highly acceptable to patients.

In particular, this study demonstrates three main outcomes. First, effectiveness was dose dependent. However, the plateau of efficacy is reached with the 100 g dose, without further improvement when the dose is increased to 150 g. In other words, there was no significant difference between 100 and 150 g, but both these doses were superior to 50 g.

Equally, tolerability analysis showed dependence on the dose, but without a plateau effect, as the frequency of AEs related to the study drug was very low with the 50 g dose, and rose as the dose increased to 100 g and then to 150 g. Surprisingly, analysis of intestinal gas levels showed no dose-dependent effect on H_2_ and CH_4_ concentrations. This finding deserves particular mention as the fear of reaching dangerous gas concentrations when mannitol is used led to it being banned as an unsafe BP product based on a few case reports. It was unusual that this issue was not further examined as mannitol proscription in Europe and the USA was based on only a few warnings and not on clinical evidence.

The current study, in contrast, has provided evidence that, after the standard procedure of washing and air insufflation, mannitol did not cause potentially dangerous levels of H_2_ and CH_4_. These gases were found only in a minority of patients, but at very low concentrations and far below the level of potential risk. Furthermore, it is important to note that H_2_ and CH_4_ concentrations were not dose dependent. This finding may encourage reconsideration of mannitol as a useful and safe laxative for BP. Moreover, the high rate of adequate bowel cleansing (94.44%) confirmed mannitol 100 g as a candidate for comparison with reference laxatives.

The high adherence rate, the ease of use, the pleasant taste, and the high rate of patients who would reuse it underlined the positive characteristics of the study drug, which potentially differentiate mannitol from the other drugs currently used for BP.

It is noteworthy that these findings were confirmed by controlling for the two stratification factors at randomization (study centre and presence of constipation in the 2 weeks before randomization) and by a model assessing the influence of potential prognostic factors (i.e., age and number of previous unsuccessful bowel cleansing procedures) on dose response.

## Conclusion

The results of this dose-finding phase II trial demonstrated that mannitol 100 g was effective for adequate bowel cleansing, safe, and well accepted by patients. Therefore, mannitol 100 g was selected as the optimal dose to be used in the subsequent phase III comparative study.

## Data Availability

The data that support the findings of this study are available from the corresponding author upon reasonable request.
